# Decoding Uncertainty Quantification for Oncology—An Illustration Using Radiomics

**DOI:** 10.3390/diagnostics16050700

**Published:** 2026-02-27

**Authors:** Florian van Daalen, Balu Krishna Sasidharan, C. Praveenraj, Amal Joseph Varghese, Andre Dekker, Leonard Wee, Rianne Fijten, Aparna Irodi, Hannah Mary T. Thomas

**Affiliations:** 1 Department of Health Promotion, Care and Public Health Research Institute (CAPHRI), Maastricht University, 6211 LK Maastricht, The Netherlands; f.vandaalen@maastrichtuniversity.nl; 2 Quantitative Imaging Research and Artificial Intelligence Lab, Department of Radiation Oncology, Unit 2, Christian Medical College Vellore, Vellore 632004, India; 3 Department of Radiation Oncology (Maastro), GROW Research Institute for Oncology and Reproduction, Maastricht University Medical Centre+, 6229 HX Maastricht, The Netherlands; 4 Department of Radiodiagnosis, Christian Medical College Vellore, Vellore 632004, India; 5 Biomedical Informatics Unit, Christian Medical College Vellore, Vellore 632004, India

**Keywords:** uncertainty quantification, aleatoric, epistemic, radiomics, thymic epithelial tumours

## Abstract

While AI models are developed in oncology for predicting different clinical outcomes, the focus is often on accuracy and many fail to adequately communicate the degree of certainty in these predictions. To improve clinical decision-making in oncology, this work introduces the idea of uncertainty quantification (UQ) for AI models using an illustrative example. Our goal is to help radiologists and oncologists better understand prediction reliability by integrating UQ. Our illustrative example is a Radiomics Risk Model (RM) for Thymic Epithelial Tumours, developed to provide a basic understanding of the mechanism to evaluate the degree to which individual patient data matches the training set. The study demonstrates the concept of measuring uncertainty in artificial intelligence (AI) models using a simple example of distance measures within the feature space and example cases where uncertainty is addressed with probable causes. The paper highlights specifically where the clinicians may need more information to improve their confidence in their AI-driven assessments for clinical diagnostics.

## 1. Introduction

Most AI models developed for radiology and oncology focus on prediction accuracy. To improve their accuracy, the first suggestion is always to increase the dataset size. However, what happens when the dataset is small, as is the case with a rare disease? This was the situation we found ourselves in at our hospital when the radiologists were interested in creating an AI-based risk stratification for Thymic Epithelial Tumours using CT images.

After we created a CT Radiomics model using the data from over 10 years, the main question the radiologists had “If I use this model to predict the risks for a new patient, how sure can we be about the prediction, given that we cannot increase the size of the dataset?” One suggested approach in this situation is to include prediction uncertainty, which matches what Kompa et al. said: ‘medical Machine learning (ML) models should have the ability to say “I don’t know” or “I am not confident enough”’; however, most current models do not have this capability [1]. Additionally, the concept of prediction uncertainty is difficult to explain to our clinical colleagues. Although there are studies on this topic, they are often too technical or do not explain it in a way that applies to our use-case. The goal of this paper is to introduce the concept of prediction uncertainty, uncertainty quantification, its relevance, and its practical implications in the clinic for radiologists or oncologists. We do not introduce any new UQ frameworks. Instead, we use a simple metric to illustrate the concept and quantify the predictive uncertainty in models and see the implementation of the metric in an illustrative example.

### What Is Prediction Accuracy and Prediction Uncertainty?

Prediction accuracy and prediction uncertainty are intrinsically linked but are two distinct concepts. The prediction accuracy of a model indicates how close the predictions were to a known target value and is often reported as an average performance over the test dataset.

Prediction accuracy compares the model’s predicted values to the known target value and represents a ratio of the correctly predicted classifications to the total test dataset. It helps to answer the question: How frequently did the model correctly classify (both True Positives + True Negatives) across all the test cases provided during model development.

Prediction uncertainty describes how much those predictions, and the corresponding target values in the data, can vary (e.g., due to measurement error, reader variability, or intrinsic biological variability).

**Figure 1:**
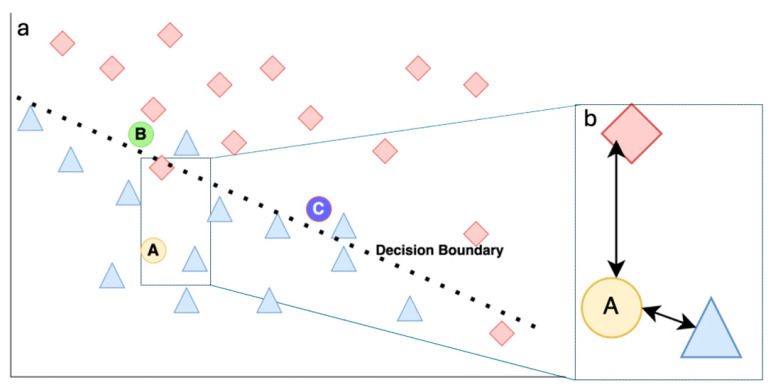
Schema explaining UQ concepts. (**a**) Training data distribution and the decision boundary as set by the model classifier after training. Representative test Cases A, B and C are shown. (**b**) Zoomed-in version showing the distances of test Case A from other examples in the training data from the two classes (Blue and Red).

Uncertainty can occur in one of two ways. One is when the data is imperfect (i.e., this uncertainty cannot be reduced by acquiring more data). Examples of the causes of this type of uncertainty are errors in measurement (e.g., weight from an uncalibrated digital weighing scale), the variability present in the image modalities (e.g., differences in images due to different reconstruction kernels for CT), or the probabilistic nature of the outcome (e.g., survival). This type of uncertainty is called aleatoric uncertainty. The second type of uncertainty occurs due to limited or unrepresentative training data (common in rare diseases) or how a particular model (e.g., classification model) behaves (see Figure 1 for an overview). As such, when we begin building a model, we can assume that there is maximum epistemic uncertainty but that it can be reduced when we have a better understanding about the data, more data, better parameters, or utilize a more suitable classifier [2]. This second type is called epistemic uncertainty.

**Somekey terms and their explanations** **Machine Learning (ML)**—A subset of AI involving algorithms that learn patterns from data and improve performance on a task without being explicitly programmed for each scenario. ML methods are commonly used in risk prediction, image analysis, and clinical outcome modelling. **Features:** The variables that contribute towards the model’s output. **Feature space** represents a multi-dimensional space where each dimension corresponds to a specific feature of the given data. Every patient represents a unique data point in feature space. The dimensionality of the feature space corresponds to the number of features in the model. ***Example:*** *Imagine plotting patients on a graph where one axis is age and the other is blood pressure; age and blood pressure are features, the 2D graph is the feature space, each patient is a **data point** on it, and adding more features like heart rate or cholesterol would add more axes (dimensions) to this space.* **Distance function:** In the feature space, the notion of distance allows us to quantify how similar or different our features are collectively in a dataset. The distance between the points helps us identify clusters of similar or different classes. **Decision boundary** is typically an equation (a line, curve, or surface) that uses several features together (e.g., age + blood sugar + BMI), and the model decides based on which side of that surface a data point lies. For complex ML models (e.g., decision trees, random forests, deep nets), the boundary can be an irregular, wiggly surface, but conceptually it is still “the place where the prediction flips from one class to another”. The algorithm learns to draw this boundary from the data and is not random.

The rest of the paper will focus on epistemic uncertainty, which will be addressed as uncertainty for the remaining discussions.

A simple, intuitive way to estimate uncertainty is to ask: **How close is this new patient’s data (test data) to the examples the model has learned from training data.**

**Figure 2:**
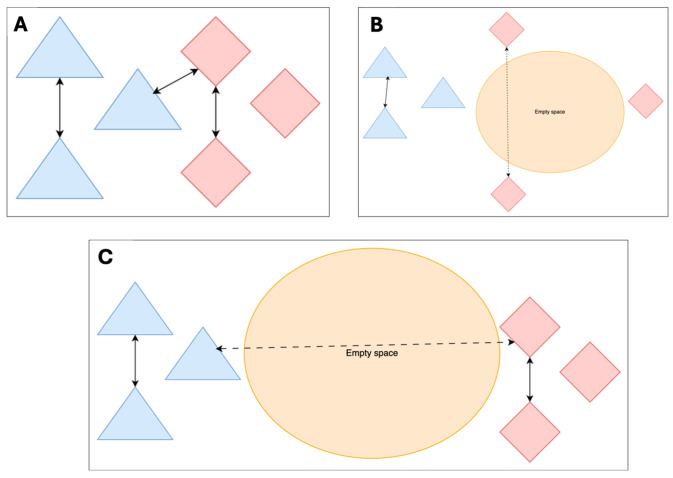
(**A**–**C**): Representative images explaining the distances in the feature space. For visualization purposes, continuous arrows represent shorter distances than the dashed arrows. Two colours show two representative feature classes.

For quantifying uncertainty, a simple method is to check if the patient’s features lie close to many similarly labelled cases that it has seen in the training set, which indicates that this new patient is like these cases contained in the training set. (Figure 1 illustrates this idea). If the feature is ‘close‘, making the uncertainty ‘low’, the prediction is more likely to be reliable. However, if the patient’s features are far from known examples (i.e., it falls into “empty” regions of feature space) (see Figure 2B,C), the uncertainty should be flagged as high [3]. This “distance to familiar territory” provides clinicians with a tangible sense of whether the model is extrapolating.

Let us consider a classification model, predicting two classes: Red and Blue. See Figure 1a for a representative logistic regression model that can do this classification. Each Red and Blue legend represents an individual data point as available in the training data. Everything above the boundary is classified as class “Red”, while everything below the line is classified as class “Blue”. For the three new cases (test data that were not part of the training), the model has classified Case A as belonging to the class “Blue” and B and C cases as “Red”.

Let us now see how we can assign uncertainty to these predicted classifications. Since A clearly belongs to the class “Blue” based on which side of the decision boundary it lies, and the distance from the ‘Blue’ example is smaller than its distance to the nearest Red example, the uncertainty is “Low” (see Figure 2B). However, when we observe B, although it is classified correctly as Red, it has a higher uncertainty since it is close to the border. Similarly, C is also close to the border, and in addition to this it is also closer to the “Blue” examples than to the “Red” examples in the training set and will thus have the highest uncertainty.

Given this background, in the next section we will use an example to see these concepts apply to our particular use case.

## 2. Materials and Methods

### 2.1. Illustrative Example

We use a Radiomics Risk Model (RM) for Thymic Epithelial Tumours, as an illustrative example, to provide a basic understanding of uncertainty quantification and to explain how the mechanism can be applied for an individual patient, given the predictions based on the data used for training the model [4].

### 2.2. Background Information About the Example Model

A Radiomics model was trained to classify patients with Thymic Epithelial Tumours into two classes of WHO-defined risk grades (i.e., low-risk (A, AB, B1) and high-risk (B2, B3) thymic epithelial carcinoma). Further description about the model development can be found in [4]. Briefly, the model used Radiomics features from the CT images of 132 patients treated between 2010 and 2024; 85 of them were low-risk and 47 high-risk WHO grades. Of these, 100 patients were used for training the model and 32 were included in the test set. We used a logistic regression classifier to build this model. Based on the percentage of high-risk patients (36%) available in the training cohort, we set the probability cut-off threshold to 0.36, which helps to set the decision boundary for this model. This threshold was selected based on the work of van den Goorbergh et al. to mitigate the class imbalance present in training data [5]. The output of this model is a ‘risk class’, with the probability of the patient belonging to the high-risk (HR) class given as a percentage. The final Radiomics model uses two features, Sphericity and the 90th Percentile Intensity; other parameters were not found to be contributing factors. The prediction accuracy, which is the proportion of all predictions that are correct at a chosen decision threshold (here a class probability ≥0.36 was determined as ‘high risk’ based on the event rate of high risk in the dataset), was 0.75 (95% CI: 0.66–0.91). This means that 75% of the time, the model was able to accurately determine if the patient who was high risk is classified correctly as high risk.

With the two features in the final model, the **feature space** becomes two-dimensional. This means that every patient can be represented as a point with two values: one for Sphericity (x-axis) and one for 90th Percentile Intensity (y-axis).

For determining the uncertainty, we use a simple distance function called Manhattan distance [6] to represent the distances in our feature space. This distance function was chosen because it is the simplest distance metric to calculate but serves well to illustrate the principle behind using a UQ.

(1) $$Distance=\left\{D_{LR}\left(y\right),\;D_{HR}\left(y\right)\right\}$$

For the two classes we are using (high risk (HR) and low risk (LR)), we would represent how an individual patient with a label ‘HR’ should be close to other individuals in the training data with label ‘HR’ and far away from individuals with the label ‘LR’.

If we find that the median distances between the two LR and HR groups are equal (see Figure 2A), we can assume that the inter-group median would also be equal, indicating that all the training cases are roughly equally far apart from each other. This represents a perfect scenario in which the entire feature space is evenly covered and thus requires a high number of samples to be collected relative to the size of the feature space. Additionally, with a complex feature space with more features and hence more dimensions, and depending on the underlying feature distribution, this relatively even distribution of samples may be limited to sub-sections of the feature space. As such, this scenario is unrealistic to hold for the entire feature space.

In Figure 2B, if we observe that the distances in the LR group are smaller than the distances seen in the HR group, it could be interpreted that LR examples are more clustered together than the HR examples in the training dataset. This indicates there is potentially empty space in the feature space that the HR examples cover.

Similarly, in Figure 2C, if the HR distances and LR distances are both equal but are less than the inter-group distance, we can assume that the two groups are clustered in a similar manner, but the two clusters themselves are far apart. This also implies that parts of the feature space are empty because there are no examples in the training set that are available there.

### 2.3. Uncertainty Quantification

Uncertainty quantification involves systematically evaluating the uncertainty in predictions made by models. Here, we used distance metrics to help quantify the dissimilarity between the test/evaluation/holdout data and the training data the model is based on, providing simple interpretations of the distances in the feature space.

We measure the uncertainty for individual patient ‘*y*’ for a particular label *x* using the equation.

(2) $$Uncertainty\left(y,x\right)=D_{x}\left(y\right)*\left(\frac{D_{x}\left(y\right)}{a}\right);$$

$$a=min\{D_{LR}(y),D_{HR}(y)\}$$

a represents the distance between individual *y* and the nearest individual in the training set who does not have label *x*.

In Equation (2), *D_x_*(*y*) represents the absolute distance to label *x*. The smaller this term, the closer individual *y* is to individuals with label *x*. The term *D_x_*(*y*)/*a*) represents the relative difference between the labels. Once calculated, an uncertainty score closer to 0 is better, as this suggests that we are more certain of the model’s prediction for an individual patient. Additionally, a perfect uncertainty score of 0 indicates the training dataset contained a patient with the same features as one of its examples.

**Figure 3:**
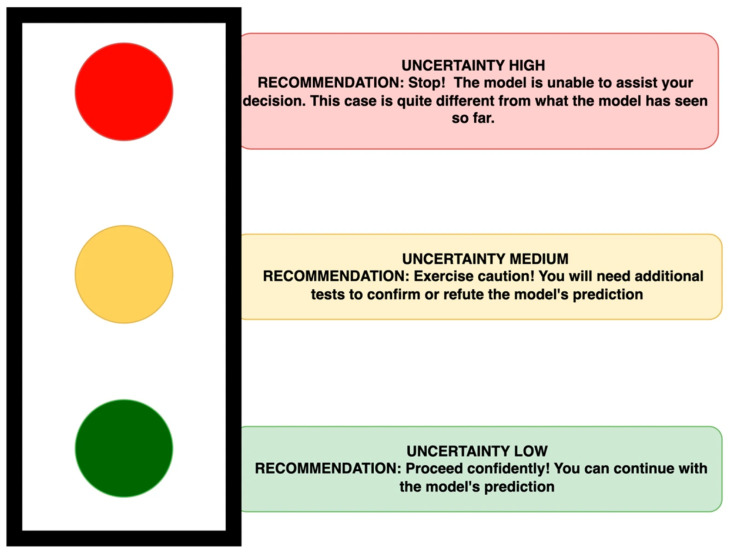
Traffic light system to assess the uncertainty level.

By presenting uncertainty alongside the prediction probability, we think it could help the radiologist to make more informed decisions. However, one significant challenge in providing individual numerical thresholds is that these values may become an artificial “magic cutoff”. This binary approach can oversimplify complex clinical scenarios, leading to decisions being made based solely on whether a number falls above or below this predetermined threshold. A classic example of this kind of approach in imaging is the PET Standard Uptake Value to separate benign from malignant lesions [7]. Here, a fixed value of SUVmax of 2.5 ignores crucial biological details and varies with scanner technology, patient factors (size, blood glucose), and reconstruction methods, leading to potential misdiagnosis. So, instead of individual scores, we suggest a traffic light systems (TLS) of recommendations which can categorize the predictions based on their associated uncertainty levels relative to the average uncertainty in each model, based on its training data (Figure 3). This means the traffic light systems can, and should, be fine-tuned for each individual model and the individual clinic. This fine-tuning is about relevant thresholds and will differ across datasets, diseases and even institutional contexts and should be calibrated for each model. The choice of appropriate thresholds should be guided by disease specific guidelines, the cost of misdiagnosis, and the choice of the type of uncertainty metric used, as different metrics measure different sources of uncertainty, etc. Hence, it is not recommended to have any generic thresholds for traffic light systems.

## 3. Results

In this section we will discuss the results of our illustrative experiment.

### Distances in Feature Space for the Training Dataset

**Figure 4:**
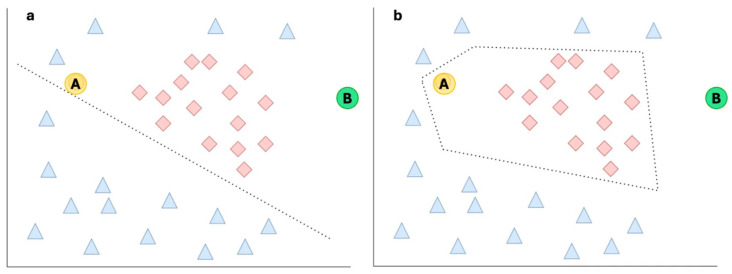
Possible distribution of data points in feature spaces and decision boundaries.

For the two risk groups LR and HR, we calculated the within-group distances of the features using Manhattan distance, and their spread is represented in Table 1. This training dataset represents a situation described in a Figure 4a. The median distances between the two groups LR and HR are almost equal (0.20 and 0.19), indicating that all the training cases are roughly equally far apart from each other.

The minimum average distances all show that HR has a smaller inter-group distance (1.12), indicating HR cases are more clustered than LR cases, as described in Figure 2B. Additionally, we see that the intra-group distance from HR to LR is smaller (1.78) than that from LR to HR (5.37), indicating that there may be ‘empty spaces’ between LR and HR. It is important to note that the minimum average intra-group distance is asymmetrical.

Now let us illustrate how the prediction probability and uncertainty scores can be used in conjunction for individual cases. In Table 2, predicted risk class presents the output of the model, with the associated probability of that case belonging to the HR class. By calculating the distance metrics, we can find how close the features in this individual case were to the nearest LR and HR examples in the training set. Based on the uncertainty quantification (Equation (2)), the scores were generated. The class with the least score closest to 0, among the two classes, determined the final risk class.

The examples in Table 2 also present possible scenarios where uncertainty could be higher and possible reasons for the same. In Example 1, the probability of the individual test case being assigned as high risk should be greater than the threshold set at 0.36. Although the true risk for this patient was LR, our model predicted it as HR. Our distance function indicates that the distance to the LR label is relatively low, whereas the distance to the HR label is relatively high. This indicates that Example 1 may be a case that is close to the decision boundary of the two classes, which leads to a high degree of uncertainty for the HR label (see Cases B and C in Figure 1a).

Examples 2 and 3 illustrate the limitations of our simple distance-based uncertainty metric. To explain these, let us refer to Figure 4. Both examples could be Case A or B and may lie in “empty” parts of the feature space, suggesting that this is where our training data does not have many examples. In Figure 4, Case A has a high uncertainty as it is far from the HR examples but is close to the LR examples. Case B shows a relatively high uncertainty, as it is far from all other examples; however, since Case B is still clearly closer to the HR class than to the LR class, it is not identified as a true outlier.

## 4. Discussion

In this article we proposed that AI tools used in clinical practice should include an uncertainty quantification measure to improve model reliability and improve trust among clinicians. We have briefly explained the principles behind using such a UQ using a simple illustrative AI model and a UQ measure. We have illustrated how this can work in practice with a small experiment based on this simple model and UQ measure.

**Figure A1:**
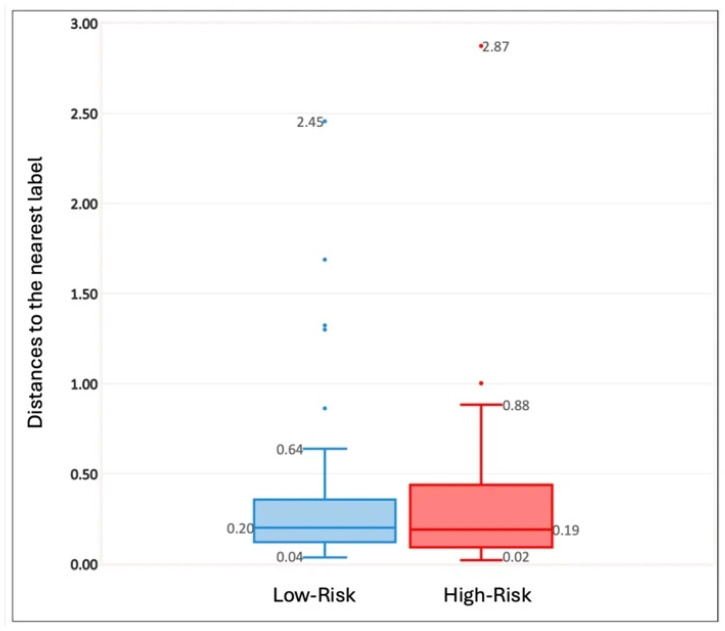
Box and Whisker plot showing the distances of the labels in the low and high-risk Groups in the training dataset.

Based on the results of this simple Radiomics model, for a new patient, the clinician could get a prediction of the patient being at risk of being in one of the two classes. However, this result does not give the clinician a sense of how certain the model is about the prediction. By including the UQ score and the information regarding the minimum, maximum and interquartile distances observed in the training dataset, we can put the UQ score into context (Table 2, Appendix A Figure A1).

However, instead of as shown in Figure 1, there is always a possibility that our feature space may even be as shown in Figure 4. In that case we can see how our simple logistic regression model may struggle and our decision boundary, which is a straight line, might be limited to classify the two classes. So, another AI model may be able to group them better, and then the shape of this decision boundary would no longer be a straight line—it could be a curve or surface (Figure 4b).

If we assume Figure 4b to be the representation of the real model, we can see what the uncertainty measure got correct, and what it did wrong. Although it correctly identified Case A as having a high degree of uncertainty with respect to LR, it failed to assign a high UQ score with respect to HR. Case B was incorrectly identified by the UQ score as acceptable, despite being an outlier, and Case A was incorrectly identified as an outlier due to the lack of examples in that area of the feature space. A better uncertainty measure, such as a weighted distance function or one that more strongly incorporates empty local feature space, could potentially have handled both cases correctly.

When the uncertainty score is high, relative to the average uncertainty in the model used, we suggest that it be treated like a situation in which we have a difficult case, where there is not a clear-cut decision. Based on the level of risk introduced by a misdiagnosis, a suggested practical step would be to get more contextual information, much like how it is acceptable practice to seek a second opinion from a colleague or specialist to either provide insights based on experience or alternative perspectives. We believe it should be no different when we employ AI-based models. This will allow us to make a more informed decision. Another strategy may be to include additional testing (such as biopsies or blood tests) which may allow us to confirm or refute the initial findings of the model and reduce uncertainty. For example, if the initial imaging suggests a high-risk lesion but the uncertainty is high, a biopsy could be ordered if the clinician thinks histopathological information might help with the diagnosis. In other situations, it may be advisable to adopt a “wait and see” approach, particularly in situations where we know the disease shows a slow progression; see an example case in the insert below.

The illustrative Manhattan distance-based UQ presented here is also a k-Nearest Neighbour-based approach, with k being 1. A simple improvement to make the UQ more robust would be the utilization of a larger k to account for individual outliers in the data. More advanced methods may help achieve better results [8]. Additionally, it is important to note the UQ used here only addresses epistemic uncertainty, as it is focused on outlier detection and is unfit in scenarios where aleatoric uncertainty is deemed highly important.

Incorporating an uncertainty metric into our model is not just about quantifying risk; it also improves the explainability for the users by providing additional insights into the inner workings of a given model. However, it is important that the users be provided training on how the AI models work. Only then will they be confident to use the AI models and gain familiarity with interpreting these metrics appropriately in their given context [9].


***Anecdotal***
***example of how this model could be used for a new patient in observational mode.***

*A 67-year-old male was evaluated for complaints of lower urinary tract symptoms for the past 3 months in June 2025. On evaluation, his Prostate Specific Antigen (PSA) was evaluated, and biopsy was reported as acinar adenocarcinoma (GS 4+3). A PSMA PET scan done in August 2025 showed locally advanced carcinoma of the prostate and a well-defined soft tissue density lesion measuring 19 mm × 28.5 mm in the anterior mediastinum abutting the ascending thoracic aorta with no fat/calcified components (incidental finding).*

*Based on these incidental findings, the thoracic lesion, which could be a thymic epithelial tumor (TET), was evaluated using the Radiomics8 risk model.*

***Output from our prediction model with UQ***
Model Predicted Risk ClassProb. of HR Class (%)Dist. to Nearest LRDist. to Nearest HRLR UQ ScoreHR UQ ScoreFinal Risk ClassLR100.210.990.044.76
**LR**

*The model predicted this patient to be low risk, with a probability of being high risk of 10%. Based on the distance metric, the patient’s features were ‘closer (0.21)’ to LR examples than the HR (0.99) examples in the training cohort. The final lower UQ score for LR (0.04) indicates that this is most likely to be LR and hence was labelled so in the final risk class.*

*Implementing the TLP (Figure 3) by using a simple threshold (>75% = uncertainty high; 50–75% = uncertainty medium; <50% = uncertainty low), this patient’s distance (LR = 0.21) when compared with Table 1 was categorized in the amber category = uncertainty medium—exercise caution.*

*The clinicians received the predicted risk and the degree of uncertainty in the classification in a two-step report. This aligned the multi-disciplinary team’s decision to go ahead with prostate cancer treatment and to take a ‘wait and watch’ strategy regarding the TET, given the patient’s age.*


Although we argue that incorporating uncertainty metrics can improve the explainability of the models, it does come with its caveats. Utilizing uncertainty correctly is highly context-dependent. What methods should be used will differ per project depending on the type of uncertainty and the sources of uncertainty, as well as the extent to which information on this uncertainty can be translated into practical actions and the risk of misclassifications. High uncertainty does not always equate to actionable insights; sometimes, it just indicates that the model lacks sufficient data in certain regions of the feature space. Just like we would not base our conclusions on the findings of a single test without taking the contextual information leading to the test, we should adopt a similar approach when incorporating AI-based predictions. It should also be noted that overreliance on uncertainty scores without clinical context and misinterpretation of uncertainty thresholds could lead to serious clinical errors [3,10]. This illustrative framework cannot be generically extended since the choice of the most appropriate UQ metric will depend on the model architecture, training distribution (feature space) and clinical stakes.

## 5. Conclusions

Uncertainty quantification may improve explainability for clinicians while implementing AI-based predictive models. However, the implementation of the models and their uncertainty metric must be accompanied by adequate training and contextual awareness. Recognizing when additional information is necessary and employing structured approaches like the traffic light systems might be useful in clinical situations, even when there is uncertainty associated with the predictions.

## Figures and Tables

**Table 1:** Summary of feature distances in the training data.

Training Dataset	Metric/From–To	LR	HR
Within-group distance distribution	Min	0.04	0.02
	25%	0.13	0.09
	50% (Median)	0.20	0.19
	75%	0.34	0.38
	Max	2.45	2.87
Mean intra-/inter-group distances	LR → LR (intra-LR)	1.69	
	LR → HR (inter-group)		5.37
	HR → LR (inter-group)	1.78	
	HR → HR (intra-HR)		1.12

**Table 2:** Example cases illustrating the predictions along with their associated uncertainty quantification (UQ) scores. The UQ scores used to determine the final risk class are highlighted in bold.

E.g. #	Predicted Risk Class	Prob. of HR Class (%)	Dist. to Nearest LR	Dist. to Nearest HR	LR UQ Score	HR UQ Score	Final Risk Class	True Risk Class (Pathology)
1	HR	48	0.19	0.28	0.13	0.42	LR	LR
2	HR	70	0.19	0.34	0.11	0.60	LR	HR
3	HR	98	3.83	0.51	28.48	0.07	HR	LR

## Data Availability

The data and model used in the study can be found in https://github.com/MaastrichtU-CDS/PracticalUncertainty (accessed on 23 February 2020).

## Abbreviations

The following abbreviations are used in this manuscript:

RM	Risk Model
TET	Thymic Epithelial Tumour
UQ	Uncertainty Quantification
WHO	World Health Organization
HR	High Risk
LR	Low Risk
IQR	Interquartile Range
AI	Artificial Intelligence
